# Wild-Type and Mutant FUS Expression Reduce Proliferation and Neuronal Differentiation Properties of Neural Stem Progenitor Cells

**DOI:** 10.3390/ijms22147566

**Published:** 2021-07-15

**Authors:** Eleonora Stronati, Stefano Biagioni, Mario Fiore, Mauro Giorgi, Giancarlo Poiana, Camilla Toselli, Emanuele Cacci

**Affiliations:** 1 Center for Translational Medicine, Department of Physiology, Temple University Lewis Katz School of Medicine, Philadelphia, PA 19140, USA; eleonora.stronati@temple.edu; 2 Dipartimento di Biologia e Biotecnologie “Charles Darwin”, Sapienza Università di Roma, 00185 Rome, Italy; stefano.biagioni@uniroma1.it (S.B.); mauro.giorgi@uniroma1.it (M.G.); camilla.toselli@gmail.com (C.T.); emanuele.cacci@uniroma1.it (E.C.); 3 Istituto di Biologia e Patologia Molecolari (IBPM), Consiglio Nazionale delle Ricerche, 00185 Rome, Italy; mario.fiore@uniroma1.it

**Keywords:** Fused in Sarcoma/Translocated in Liposarcoma (FUS/TLS), cell proliferation, cell differentiation, neural stem progenitor cells (NSPC), ALS, FUS-linked disease, neurodegenerative disease

## Abstract

Nervous system development involves proliferation and cell specification of progenitor cells into neurons and glial cells. Unveiling how this complex process is orchestrated under physiological conditions and deciphering the molecular and cellular changes leading to neurological diseases is mandatory. To date, great efforts have been aimed at identifying gene mutations associated with many neurodegenerative diseases, including amyotrophic lateral sclerosis (ALS). Mutations in the RNA/DNA binding protein Fused in Sarcoma/Translocated in Liposarcoma (FUS/TLS) have been associated with motor neuron degeneration in rodents and humans. Furthermore, increased levels of the wild-type protein can promote neuronal cell death. Despite the well-established causal link between FUS mutations and ALS, its role in neural cells remains elusive. In order to shed new light on FUS functions we studied its role in the control of neural stem progenitor cell (NSPC) properties. Here, we report that human wild-type Fused in Sarcoma (WT FUS), exogenously expressed in mouse embryonic spinal cord-derived NSPCs, was localized in the nucleus, caused cell cycle arrest in G1 phase by affecting cell cycle regulator expression, and strongly reduced neuronal differentiation. Furthermore, the expression of the human mutant form of FUS (P525L-FUS), associated with early-onset ALS, drives the cells preferentially towards a glial lineage, strongly reducing the number of developing neurons. These results provide insight into the involvement of FUS in NSPC proliferation and differentiation into neurons and glia.

## 1. Introduction

Fused in Sarcoma/Translocated in Liposarcoma (FUS/TLS) belongs to the FET (FUS- EWSR1, TAF15) family protein and was described for the first time in myxoid liposarcoma as an oncogenic fusion of the dominant negative transcription factor gene CHOP with the gene FUS [1,2]. The interest in this protein has dramatically increased since a causative link was demonstrated between heritable and de novo FUS mutations and the dominant form of amyotrophic lateral sclerosis (ALS) [3,4,5]. FUS is a ubiquitously expressed 526 aa DNA/RNA binding protein (RBP) member, mainly localized in the nucleus, although several identified mutations in its coding regions cause its cytoplasmatic mislocalization [4,5,6,7]. Notably, many of the ALS-linked mutations clustered in C-terminal, non-classical nuclear localization signal [8].

FUS is implicated in the control of several stages of gene expression [9]: FUS controls transcription, as demonstrated by chromatin immunoprecitation combined with transcriptomic approaches that allowed to identify several FUS target genes [10]; microRNAs’ biogenesis, that is reduced by FUS/TLS depletion through a mechanism involving the modulation of the activity of Drosha [11]; and pre-mRNA processing as demonstrated by genome-wide analysis showing that thousands of RNA targets are bound by FUS [12,13,14], and by the fact that FUS deletion affects levels and splicing in about one thousand mRNAs [14]. The identification of FUS mutations in familial and sporadic forms of amyotrophic lateral sclerosis (ALS) and frontotemporal lobar degeneration (FTLD) patients suggests that modifications in RNA metabolism might be an essential event in the disease pathogenesis [4,5]. Many studies pointed out that both the loss of nuclear function and the cytoplasmic gain of function of FUS lead to ALS-associated neurodegeneration in patients and in animal models [15,16].

Notably, an association between the increased expression of the wild-type form of FUS and the ALS disease has also been observed [17,18,19]. Mutations in the 3′ untranslated region (3′ UTR) of FUS, causing increased levels of FUS wild-type (WT) protein, have been identified in ALS patients and proposed to contribute to ALS pathogenesis [19]. This is strengthened by studies of transgenic mice expressing the wild-type human FUS under the control of the murine prion promoter that allows for a broad expression throughout all the neural cell types in the central nervous system, which showed ALS-like behavior and degeneration. This study suggests that high levels of human FUS trigger gain-of-function toxicity via altered autophagy-lysosome pathway and RNA metabolism function [17]. Accordingly, transgenic homozygous mice overexpressing wild-type human FUS show spinal cord motor neuron degeneration and develop progressive hind limb paralysis, followed by precocious death [18]. Notably, these mice showed increased astroglial and microglial reactivity, suggesting a causative link between motoneuron degeneration and gliosis. Accordingly, we have recently demonstrated that astrocyte-like cells, generated from mouse and human NSPCs, conditionally expressing human wild-type FUS (hWT-FUS), show enhanced expression of inflammatory genes upon treatment with the pro-inflammatory cytokine IL1β, compared with control astrocytes. Moreover, astrocytes expressing hWT-FUS promote neuronal cell death and pro-inflammatory microglia activation; this study indicates that elevated levels of hWT-FUS intrinsically affect astrocyte reactivity and drive their properties toward pro-inflammatory and neurotoxic functions, strengthening the view that a non-cell autonomous mechanism can support neurodegeneration in ALS [20].

ALS is known to be a multifactorial disorder, and one potential mechanism leading to neuronal death includes the inappropriate expression or activation of cell-cycle proteins [21,22,23,24,25]. Transgenic mice expressing a mutant superoxide dismutase 1 (SOD1) associated with human ALS (namely SOD1G37R) develop motor neuron disease. Among several mechanisms, the authors proposed a neuronal death pathway involving Cdk5 deregulation, a kinase that lies upstream of other cell cycle regulators. Deregulated Cdk5 would trigger a signaling cascade abnormally increasing protein level and activity of the regulator of the G1-S checkpoint, Cdk4, and phosphorylation of the Rb protein, leading to the activation of the pro-apoptotic Bcl-2 family member Bax, and eventually ended with apoptotic mediated motor neuron cell death [23]. Histological analysis of spinal cord, motor cortex, and sensory cortex tissues from sporadic ALS cases and age-matched controls revealed altered expression and subcellular distribution of G1 to S phase cell-cycle regulators in affected motor neurons [24]. In a follow-up study the same authors analyzed the levels of the cell cycle checkpoint tumor suppressor protein p53 and markers of cell death Bax, Fas, and caspases by immunoblot, immunohistochemistry, and double-label confocal microscopy. This study identified p53, phosphorylate Rb,caspase-3 and TUNEL labeled neurons in the spinal cord but not in the motor cortex of ALS samples, supporting aberrant cell cycle regulator expression as a neurotoxic ALS-associated mechanism [25].

In order to identify mRNA targets of FUS associated with ribonucleoprotein complexes in the cytoplasm of neurons, Colombrita and colleagues performed high throughput RNA/DNA sequencing coupled with immunoprecipitation on the mouse motoneuronal NSC-34 cell line: among other FUS targets, an enrichment in transcripts for cell cycle-related protein was found [26]. This is in line with an additional finding showing that the RNA-binding protein FUS is specifically recruited to the promoter of Cyclin D1 (CCND1) and represses its expression in response to ionizing radiation-induced DNA damage, through non-coding RNAs transcribed from multiple 5′ regulatory regions of *CCND1* [27].

In accordance with molecular data, functional analyses support a regulatory role of FUS in cell proliferation. Quantitative proteomic analysis performed on motor neuron-like cells knocked down for FUS revealed expression changes in proteins associated with multiple processes, including cytoskeletal organization, oxidative stress, energy homeostasis, and cell cycle progression; such changes are paralleled by cell cycle regulators’ impairment [28].

Notably, proliferation of glial cells was observed by stereotaxically injecting adenovirus-encoding shFUS in the marmoset cortex; as glial activation occurred eight weeks post-injection without neuronal loss, glial cell expansion might be directly linked to a FUS regulatory function in astrocytes [29]. Furthermore, altered FUS levels modulate cell proliferation in various tumor cells [30,31,32]. Particularly, gain- and loss-of-function approaches demonstrate that FUS inhibited prostate cancer cell growth in vitro and FUS overexpression in tumor xenografts led to strong tumor regression. Progression of prostate cancer is androgen receptor-mediated pathway-dependent, and a proteomic screening indicates that androgen stimulation down-regulates FUS [30]. This study suggests that FUS can act as tumor suppressor, by reducing the levels of proliferative factors such as cyclin D1 and Cdk6 and by increasing levels of the antiproliferative Cdk inhibitor p27. However, FUS’ role in cancer cells remains puzzling and clearly dependent on the tumor origin. In fact, it has also been shown that siRNA-induced FUS downregulation in human neuroblastoma cell lines had tumor-suppressive effects [32], and the physical interaction of FUS with the long non-coding RNA NEAT1 promotes cell survival, while FUS reduction triggered apoptotic cell death in breast cancer [31]. All this evidence suggests an important role for FUS in regulating cell cycle progression and survival in several malignancies.

In light of the widely recognized role of FUS mutations in neurodegenerative diseases such as ALS and FTLD, it is important to shed new light on FUS function also in neurophysiological processes. Taking into account our limited knowledge about the role of FUS in neural cell proliferation and differentiation, we addressed these points by using a neural stem progenitor cell culture system.

In the current study, we engineered NSPCs isolated from the spinal cord of mouse embryos at E13.5 developmental stage with a plasmid, allowing for the pharmacologically regulated expression of hWT-FUS or the mutated gene FUS P525L [11,20,33]. Here, we demonstrate the critical role of FUS on self-renewing cells and neuronal commitment. We show that gain-of-function of FUS has a growth-inhibitory effect on NSPCs, regulating the expression of a specific kinase family inhibitor: CDKN1B/p15. Additionally, during differentiation, we demonstrate an anti-neurogenic effect of the hWT-FUS expression.

We also explored the effect of the expression of a mutant form of FUS, associated with the early onset of ALS in humans, namely FUS P525L, on NSPC properties. We demonstrated that its expression produced effects (e.g., decreased proliferation and impairment of NSPC neurogenic potential) comparable to human WT-FUS expression, indicating that FUS plays a role in the control of expansion and differentiation capabilities of NSPCs. Here, we show the importance of the balance of FUS expression during development, as any changes in FUS levels can affect NSPC competence to proliferate and properly differentiate.

## 2. Results

### 2.1. Expression and Localization of HumanWT-FUS in NSPCs

With the aim of understanding the effect of FUS in undifferentiated cells of neural origin, we isolated NSPCs from embryonic mouse spinal cord and established cell lines allowing for the pharmacologically regulated expression of human WT-FUS protein.

Spinal cord-derived NSPC cell lines had been previously isolated in our laboratory and demonstrated to retain neural stem cell properties in vitro even after prolonged expansion [20,33,34]. Supplementary Figure S1 shows that murine spinal cord-derived NSPCs express neural markers and upon growth factor deprivation differentiate into neurons and glia in accordance with their multipotent properties. Spinal cord-derived NSPCs represent a useful system to assess at the cellular and molecular levels the role of this ALS-associated gene and unveil its role in the early stages of CNS development.

**Figure 1:**
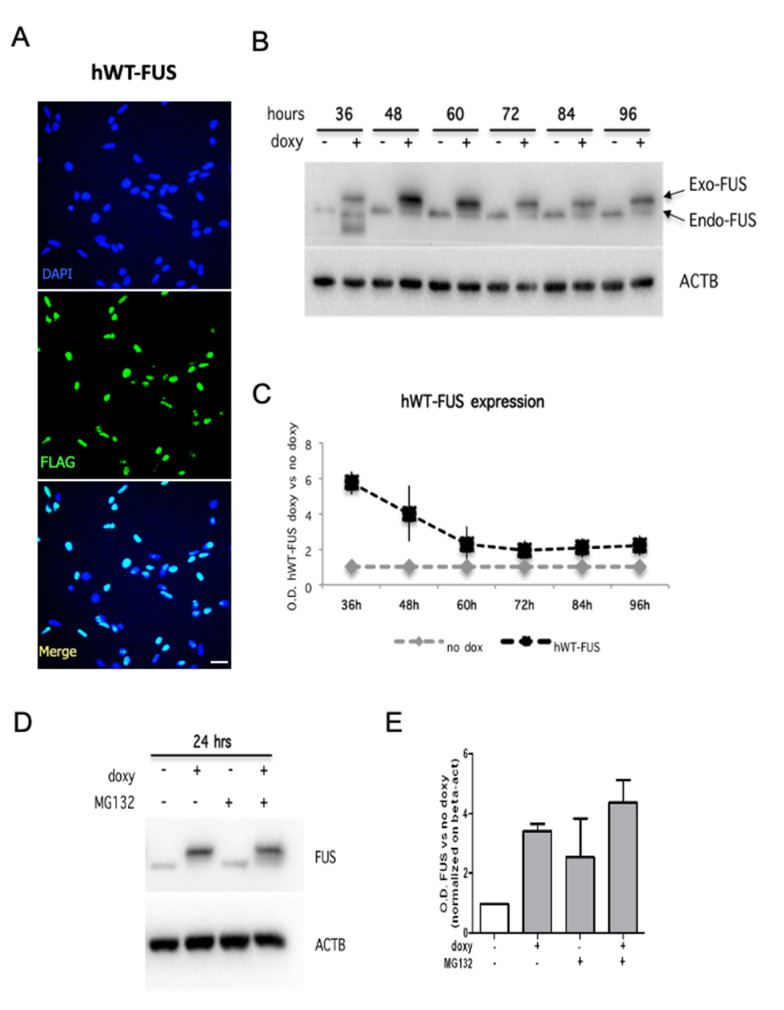
Human WT-FUS is expressed in mouse NPSCs. (**A**) Immunostaining using an anti-FLAG antibody on proliferating NSPCs electroporated with the hWT-FUS plasmid, showing the nuclear localization of the exogenous protein. Cell nuclei are stained with the nuclear dye Hoechst. Scale bar 30 µm. (**B**,**C**) Representative immunoblot of proliferating NSPCs in time course between 36 and 96 h from transgene induction (**B**) and densitometric analysis of hWT-FUS transgene expression (**C**). (**D**,**E**) Representative immunoblot of proliferating NSPCs grown for 48 h and then treated with MG132 (0.2 uM) for 24 h and densitometric analysis of FUS levels. Beta-actin is used as a loading control. Values are expressed as the average of three independent experiments. Data are presented as a mean ± SEM.

We generated an inducible cell line in which human WT-FUS expression was pharmacologically activated by doxycycline administration. As a control, we generated NSPCs expressing the red fluorescent protein under the control of a doxycycline-regulated promoter (RFP-NSPCs). We found that doxycycline treatment did not affect RFP-NSPC expansion and differentiation capabilities, indicating that the used concentration of this drug does not exert off-target effects on NSPCs (Supplementary Figure S2). Immunocytochemical detection showed that exogenous FUS is localized in the nucleus of virtually all transfected cells (Figure 1A), as well as the endogenous protein. We quantified human WT-FUS (hWT-FUS) protein at different time points upon doxycycline administration, showing that hWT-FUS protein was strongly expressed at 36 h and remained elevated up to 48 h, decreasing at subsequent time points, with an idle point at 72 h (Figure 1B,C). These data suggested a physiological control of the expression of the exogenous protein, probably due to the autoregulatory feedback system [35,36].

As an alternative regulatory system of exogenous FUS expression in NSPCs, we hypothesized the involvement of the proteasome. To explore this possibility, we used MG132 (benzyloxycarbonyl-leucyl-leucyl-leucinal) to inhibit the ubiquitin proteasome system (UPS) [37,38]. MG132 treatment for 6 and 24 h appears to partially counteract the degradation of hWT-FUS protein, suggesting that UPS operates a control of the amount of hWT-FUS expression, although this system is not exclusive (Figure 1D,E).

### 2.2. Expression of hWT-FUS Protein Markedly Decreases Cell Growth

**Figure 2:**
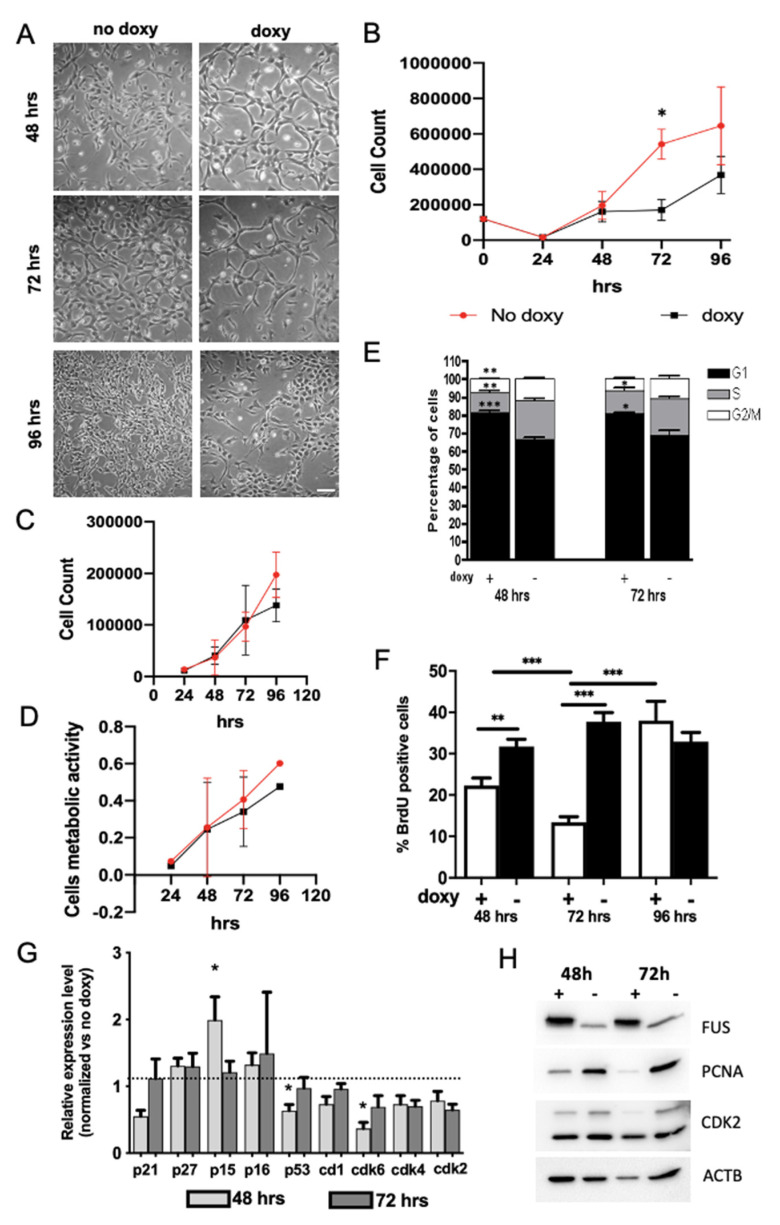
Expression of hWT-FUS reduces cells’ growth, inducing an arrest in G1/G0 phase in NSPCs. (**A**) Representative bright-field images of proliferating NSPCs between 48 and 96 h, showing a reduction of cell number in induced cells compared to control at 72 h. Scale bar 30 µm. (**B**) Growth curve of NSPCs expressing or not hWT-FUS between 24 and 96 h. Note that cell number significantly differs between doxycycline-treated and untreated cells. (**C**,**D**) Graphs show quantification of the NSPC cell death assessed by trypan blue exclusion test of viability (C) and NSPC metabolic activity, measured with MTT assay (**D**) between 48 and 96 h. (**E**) FACS analysis of proliferating NSPCs treated with or without doxycycline for 48 or 72 h. (**F**) Analysis of BrdU incorporation. The cells are stained after a pulse of 1 uM of BrdU for 20 min at the indicated time points and the number of BrdU+ cells is quantified. Nuclei are stained with Hoechst. (**G**) Graph shows qPCR of G1/G0-related gene expression levels in hWT-FUS NSPCs maintained under proliferative conditions for 48 or 72 h. Values are expressed as a fold change compared to the expression of the same genes in the uninduced cells. (**H**) Representative immunoblot of whole cell lysates obtained from NSPCs treated with or without doxycycline for 48 or 72 h. FUS protein was revealed using monoclonal anti-FUS antibody. Actin is used as a loading control. Data are presented as a mean ± SEM of 4 independent experiments. Student’s t-test. * *p* < 0.05 ** *p* < 0.01 *** *p* < 0.001.

Given the well-known role played by FUS protein in tumorigenesis and the control of tumor cells growth [30,39,40], we set out to investigate its role in the control of NSPC properties. Light microscopy revealed that hWT-FUS expression results in a strong reduction in NSPC cell number after 72 h of doxycycline administration (Figure 2A). In order to quantify this reduction, we performed a growth assay in a time course between 24 and 96 h. Non-induced cells showed an exponential growth that was not paralleled by cells overexpressing hWT-FUS. Particularly, we observed 392,000 ± 53,200 cells corresponding to a 35% reduction in cell numbers between 48 and 72 h. Interestingly, at the subsequent time point, the induced NSPCs restored their growth rate, compared to control (Figure 2B). Cell viability and metabolic activity, analyzed by trypan-blue exclusion method and MTT assay, respectively, are not influenced by transgene expression at any time point analyzed (Figure 2C,D).

Since these data strongly indicate that proliferation was altered in doxycycline-treated cells expressing hWT-FUS, we performed cytofluorimetric analyses to study the cell cycle. In agreement with previous observations, hWT-FUS NSPCs showed an increased number of cells in G1/G0 phase at 48 and 72 h, respectively, of 14.98 ± 1.18 and 12 ± 2.75, with a concomitant reduction of cells in S (9.09 ± 1.97, corresponding to ~10%) and G2/M (4.4 ± 1.44, corresponding to ~8%) phases (Figure 2E). To gain further insight, we used Bromodeoxyuridine (BrdU) to mark the cohort of dividing cells in S phase. As expected, we observed a significant reduction of the percentage of BrdU-positive cells after 48 and 72 h, respectively, of 7.39 ± 3.5 and 24.39 ± 2.52. At 72 h hWT-FUS NSPCs, in agreement with the increased cell number previously shown, restored their replicative capacity with a proliferation index similar to control (Figure 2F). This growth rescue, due to the overcoming of checkpoint G1/S, suggests an involvement of a control of FUS protein concentration to maintain cells in a healthy condition.

### 2.3. FUS Regulates the Expression of Cell Cycle Factors

To further investigate the relationship between FUS expression and cell cycle progression, we analyzed the modulation of expression of cell cycle regulators acting at G1/S checkpoint, which could therefore explain the observed cell accumulation in G1.

Although it has been demonstrated that FUS binds, via non-coding RNA, to the regulatory regions of cyclin D1 gene, blocking its transcription [27], we did not observe any changes in cyclin D1 at the time points analyzed by real-time PCR, indicating that elevation in FUS levels can impair NSPC proliferation by a cyclin D1-independent mechanism. Slight changes were observed in the levels of kinase inhibitors of Cip-Kip family members (p21 and p27), known to be regulated by FUS in prostate cancer cell lines [30]. Conversely, at 48 h we observed the increased expression of CDKN2B (p15), another member of the kinase family inhibitors, which returned to control levels at 96 h. This p15 increase has been correlated with the reduction of CDK6 expression at the same time points (Figure 2G), while no changes were observed for CDK4 expression.

These data are in good agreement with the restoration of normal growth rate and with the increased number of BrdU-positive cells 72 h after transgene induction. We further analyzed protein expression of some cell cycle progression regulators, such as CDK2 and PCNA, both involved in the transition G1/S, and we observed that both levels were strongly decreased at 48 h, in response to FUS; the expression of CDK2 showed a recovery at 72 h, while PCNA levels remained unchanged (Figure 2H). Altogether these data suggest that FUS expression controls the progression of cell cycle to S phase through the activity of p15.

### 2.4. Human WT-FUS Expression Has an Anti-Neurogenic Effect

Having demonstrated that human WT-FUS expression acts as a repressor of mouse NSPC growth, we extended our analysis by evaluating the role of hWT-FUS on NSPC differentiation. It has been shown that the physiological expression of FUS seems to be reduced during differentiation of cortical neural progenitor cells by miR-141 [41]. Upon growth factor depletion and in the presence of DAPT (see Material and Methods), a gamma secretase inhibitor affecting Notch signaling activation, NSPCs undergo differentiation into a cell population enriched in neurons [20,42,43].

**Figure 3:**
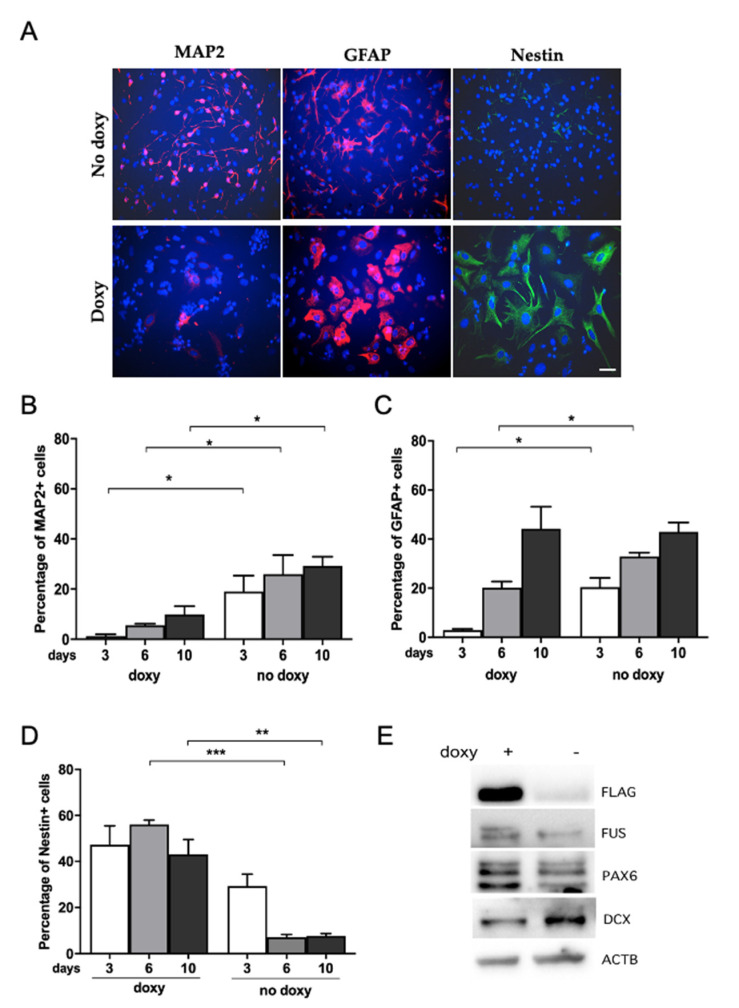
hWT-FUS expression reduces neuronal differentiation. (**A**) Representative images of NSPCs maintained in neuronal differentiation medium (DAPT 0.5 nM) for 3, 6, and10 days and then fixed and stained for the neural marker Nestin, the glial marker GFAP and the neuronal marker MAP2. Scale bar 30 µm. (**B**–**D**) Percentage of cells positive for MAP2 (**B**), GFAP (**C**) and Nestin (**D**) at the analyzed time points. (**E**) Representative immunoblot of the indicated proteins in NSPCs differentiated for 6 days. Note an increase in PAX6 level and a decreased level of the immature neuronal marker DCX with respect to doxycycline-untreated cells. Beta-actin is used as a loading control. Data are shown as mean±SEM of three independent experiments. Mann–Whitney test * *p* < 0.05 ** *p* < 0.01 *** *p* < 0.001.

In order to quantitatively evaluate the effect of hWT-FUS expression on neuronal differentiation, we determined by immunoblot the expression of neuronal marker DCX and neural progenitor marker PAX6 on NSPCs differentiated for 6 days in the presence of doxycycline, compared with non-induced cells. Interestingly, we observed a reduction of DCX expression and an increased expression of PAX6, suggesting that hWT-FUS expression counteracts neuronal differentiation (Figure 3E). We further analyzed, by immunocytochemistry, changes in cell population distribution following NSPC differentiation for 3, 6 and 10 days. The percentage of differentiating neurons, identified by using the neuronal marker MAP2, was strongly reduced (15.84 ± 3.04, corresponding to a reduction of about 35%) in doxycycline-treated cells when compared to control at all the time points analyzed (Figure 3A,B). This reduction suggests that expression of hWT-FUS interferes with neuronal commitment (and/or survival).

On the other hand, we observed also a decrease in the percentage of GFAP-positive cells in cultures expressing hWT-FUS compared to control after 3 and 6 days of differentiation, although after 10 days the percentage of GFAP-positive cells is similar between induced and non-induced cells (Figure 3A,C). Interestingly, the analysis showed that NSPCs treated with doxycycline maintain the expression of neural progenitor marker Nestin under differentiating conditions, with an increase of 45.87 ± 1.83 corresponding to about 18% after 6 days, compared to control (Figure 3A,D). As Nestin is downregulated upon differentiation and is replaced by neuronal or glial-specific intermediate filament proteins [44,45], our data might indicate that expression of hWT-FUS maintains NSPCs in an undifferentiated state.

To further shed light on the effect exerted by hWT-FUS expression on NSPC neuronal differentiation, we quantified by immunostaining the number of cells expressing Nestin, MAP2 and GFAP over time. NSPCs were maintained in proliferating conditions for 48 h to obtain the highest transgene expression. We verified the expression of the transgene and of the stem cell marker Nestin at this time point. After 48h, we induced neuronal differentiation and analyzed the marker’s expression at 1, 3 and 6 days of differentiation.

**Figure 4:**
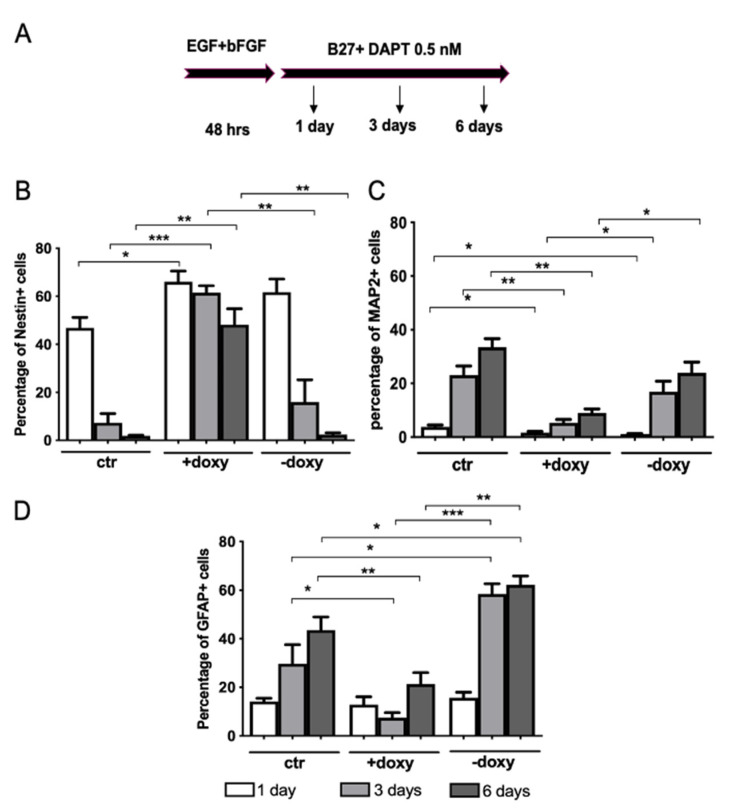
Neuronal differentiation of NSPCs is impaired by the expression of hWT-FUS. (**A**) Schematic representation of the procedure used to quantify the neuronal, glial and undifferentiated population obtained upon 6 days of differentiation. NSPCs were maintained in expansion medium (EGF + bFGF) supplemented with or without doxycycline for 48 h. The cells were then switched to neuronal differentiation medium (B27 + DAPT 0.5 nM) for 24 h, 3 days and 6 days. (**B**–**D**) Quantification of the percentage of stemness marker Nestin (**B**) and differentiative markers, respectively neuronal MAP2 (**C**) and glial GFAP (**D**), immunopositive cells after 1 day, 3 days and 6 days. The NSPCs ”+doxy” were maintained with doxycycline both under proliferative and differentiative conditions, while ”-doxy” where exposed to doxycycline only during the first 48 h (proliferative condition). Data are presented as a mean ± SEM of 3 independent experiments. Mann-Whitney test * *p* < 0.05 ** *p* < 0.01 *** *p* < 0.001.

With the continuous induction of hWT-FUS by doxycycline treatment (+doxy) cells maintained a high expression of Nestin (46.33 ± 3.94), associated with a strong reduction of the neuronal marker MAP2 (24.48 ± 2.95) (Figure 4B,C), relative to uninduced cells (CTR). Interestingly, in the ‘wash-out’ experiment, while the doxycycline induction is maintained only during the 48 h of proliferative condition (-doxy), the number of Nestin-positive cells returned to baseline level within 3 days, compared to CTR cells (Figure 4B). This reduction was associated with an increased expression of GFAP-positive cells and a modest enhancement of MAP2-positive cells (Figure 4C,D). We have also verified the reduction of transgene expression in NSPCs -doxy, observing that hWT-FUS level returned to baseline within 3 days.

Taken together these results suggest an involvement of FUS during neuronal differentiation, at the very early stages of NSPC commitment. Notably, in this differentiation process, the amount of WT-FUS expression is the key: in the ‘wash-out’ experiment we observe a delay in the differentiation of NSPCs and a switch to glial lineage, compared to control and to continuously induced cells, due to the removal of the exogenous transgene expression. These data suggest that WT-FUS has a dual role at the earliest stages of neuronal differentiation: on the one hand it has an anti-neurogenic effect; on the other hand, it maintains NSPCs in an undifferentiated state.

### 2.5. Expression of Mutant Human FUS Arrests Proliferation and Promotes Glial Differentiation

To get insight into the development of FUS-linked disease, we investigated the effect of the expression of one mutant form of FUS (P525L-FUS), associated with early-onset ALS, in the maintenance and differentiation of NSPCs. In yeast, Kryndushkin and colleagues have demonstrated that the expression of mutant FUS results in its massive aggregation inducing cellular toxicity and motoneuronal death [46].

**Figure 5:**
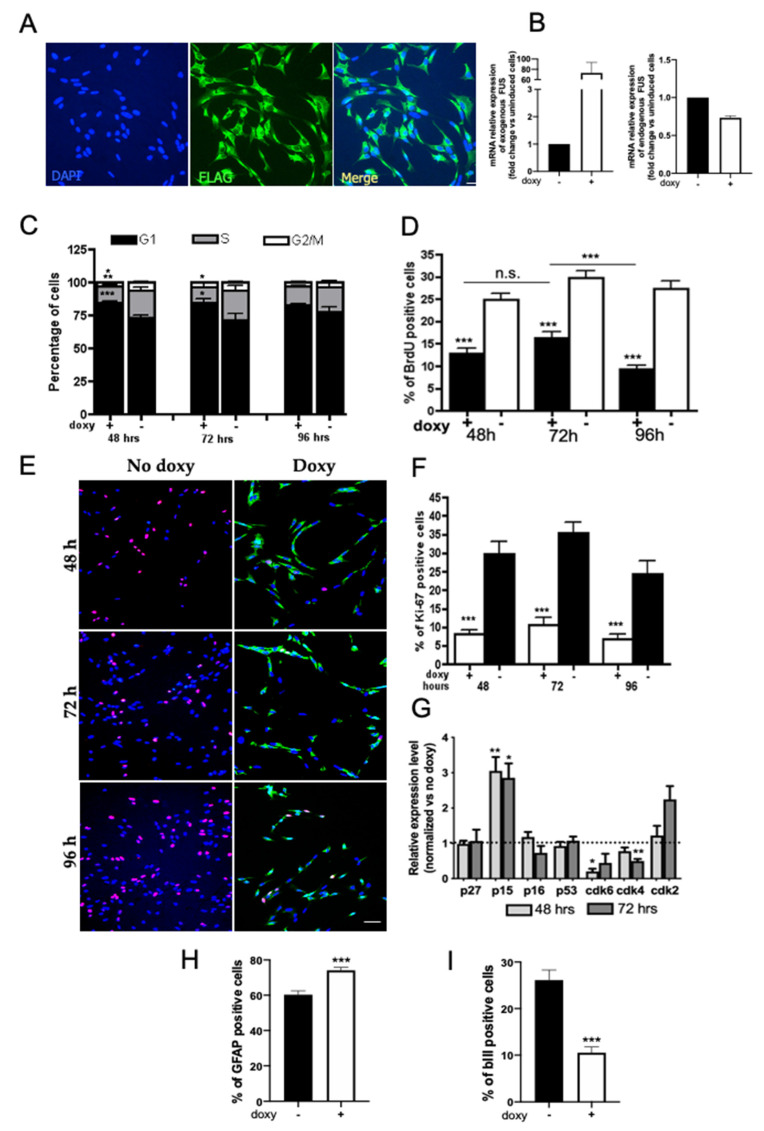
Mutant FUS expression impairs proliferation and neuronal differentiation of NSPCs. (**A**) Representative immunofluorescence images of proliferating NSPCs expressing mutant hFUS (green) after 48 h in the presence of doxycycline. The exogenous protein, detected by an anti-FLAG antibody, shows a mainly cytoplasmic distribution, as expected for the mutated protein. Cell nuclei are stained in blue. Scale bar 30 µm. (**B**) RT-PCR quantification of transgenic and endogenous FUS expression in proliferating NSPCs. Graphs show that human mutant FUS is strongly induced by doxycycline administration while endogenous FUS expression remains unchanged. (**C**) Cytofluorimetric analysis of proliferating mutant FUS NSPCs at 48, 72 and 96 h from transgene induction. (**D**) Quantification of the percentage of BrdU-incorporating cells. NSPCs were grown in expansion medium supplemented with or without doxycycline and analyzed between 48 and 96 h. (**E**) Representative images of NSPC maintained for 48, 72, and 96 h in expansion medium supplemented with or without doxycycline and immunostained for Ki67 (red) and the transgene mutated protein (anti-FLAG, green). Scale bar 30 µm. (**F**) The graph represents the quantification of Ki76-positive cells at the analyzed time points. (**G**) Graph shows qPCR of G1/G0-related gene expression levels in mutant FUS NSPCs’ proliferating cultures at 48 and 72 h. The values are expressed as a fold change compared to the expression of the same genes in the uninduced cells. (**H**,**I**) Percentage of neurons (βIII-tubulin^+^) (**H**) and astrocytes (GFAP^+^) (**I**) obtained from mutant FUS NSPCs differentiated in the neuronal differentiation medium for 12 days. Data shown are the mean ± SEM of four independent cell culture preparations. Mann–Whitney test * *p* < 0.05 ** *p* < 0.01 *** *p* < 0.001.

Differently from hWT-FUS, mutant FUS was localized in the cytoplasm (Figure 5A). Real time RT-PCR quantification of transgenic and endogenous FUS expression in proliferating NSPCs revealed that human mutant FUS is strongly induced by doxycycline administration, while endogenous FUS expression remains unchanged (Figure 5B).

We analyzed the effect of human P525L-FUS on NSPC cell proliferation in a frameshift from 48 to 96 h, observing a significant increase in the percentage of mutant FUS cells in G0/G1 phase (about 80%) compared to control cells, with a concomitant decrease in the proportion of cells at S and G2/M phases (Figure 5C). The G1/G0 phase accumulation was also demonstrated by BrdU immunostaining quantification (Figure 5D), confirming the inhibition of cell cycle progression through phase S. Furthermore, immunostaining for Ki67, a cellular marker of proliferation, confirmed the marked decrease in proliferating cells, with a reduction of 26.24 ± 3.99 (about 30%) of Ki67-positive cells in induced cells when compared to control (Figure 5E,F), indicating that the expression of mutant FUS determines cell cycle arrest. To explore the potential mechanism responsible for the observed reduction in cell growth, the expression of critical regulators of G1/S transition (CDK6 and CDK4 and inhibitors p27kip1, p15 and p16) was analyzed by real-time PCR. The expression of cyclin-dependent kinase (CDK) inhibitor p15 was markedly increased after 48 and 72 h of transgene expression compared to control cells, associated with a reduction in CDK6 expression; there was no significant change in the expression of CDK4 and other CDKIs analyzed (Figure 5G). In this respect it is important to take into account that literature data show that the single CDK inhibitor p15 action can induce the quiescent phase of the cell cycle (reviewed in [47]).

Based on the effect of WT-FUS in regulating NSPCs’ properties, such as differentiation, we analyzed the possible role mediated by the expression of mutant FUS in NSPCs’ neuronal differentiation. By immunofluorescence analysis of NSPCs, induced to differentiate for 12 days in neuronal differentiation medium, we observed a marked reduction in the percentage of cells expressing the neuronal marker βIII tubulin and a parallel increase in the percentage of cells expressing GFAP (Figure 5H,I). These data indicate that perturbation of the expression of FUS had an anti-neurogenic effect, in favor of the specification of glial lineage.

## 3. Discussion

Genetic alterations of FUS, a multifunctional nuclear protein involved in several steps of RNA metabolism, can be associated with human diseases, such as cancer and neurodegenerative disorders [7,9,30,48,49]. Recently, it has been suggested that changes in the balance of protein concentrations in the nucleus and the cytoplasm can provide a hint for understanding ALS pathology [17,18,19], suggesting that FUS levels are crucial for cell homeostasis. Previously, four mutations in the 3-UTR of human FUS have been identified in 420 patients negative for mutations in the currently known ALS-associated genes. These mutations cause widespread wild-type FUS accumulation linked to severe pathological outcomes [19]. Accordingly, transgenic mice overexpressing human wild-type FUS in a dose-dependent manner develop ALS with aggressive phenotype, characterized by an early onset of several pathological features, such as tremor and locomotor impairment, followed by paralysis and ultimately death [18]. This evidence suggests that FUS levels are crucial for cell homeostasis and that not only is mutant FUS toxic to motoneuron survival, but also that the increased expression of the wild-type form is sufficient to cause neurodegeneration [20].

In the present study we show the effect of the expression of human WT-FUS, or the expression of mutant FUS-P252L, associated with juvenile ALS, on growth and differentiation of NSPCs. Interestingly, it has been previously described both in human patients [19] and in mouse models [18,50] that an increased expression of wild-type FUS protein determines the development of ALS-like disease characterized by an early onset of several pathological features, such as tremor and locomotor impairment, followed by paralysis and ultimately death. These data suggest that not only is mutant FUS toxic to motoneuron survival, but also that the increased expression of the wild-type form is sufficient to cause neurodegeneration [51].

Several groups have investigated the effects of FUS knockdown in both neuronal terminally differentiated [52,53,54] and proliferating cells [10,12,55,56,57], showing that this RNA-binding protein exerts an effect on cell health. Our work reports a novel effect due to the expression of wild-type and mutant FUS on neural stem cell properties, regulating their proliferation and neuronal differentiation capability. We show that the exogenous and inducible expression of human WT-FUS strongly reduced spinal cord NSPCs’ growth, inhibiting the progression of the cell cycle at the G1/S checkpoint. This effect is time-dependent: these cells are able to overcome this arrest after 96 h in vitro and restore their proliferation, due to the controlled expression of the exogenous protein, and in turn to the autoregulatory control of the protein itself in the nucleus [35]. We did not observe in these cells the delocalization of wild-type FUS protein in the cytoplasm shown in in vivo experiments [18], suggesting that in this cellular model the exogenous expression level of the protein may be regulated by the known autoregulatory mechanism. The endogenous expression remained substantially unchanged during the analyzed time course.

Analysis of cell cycle regulators revealed that altered levels of FUS are associated with changes in the expression of several factors involved in G1/S transition of cell cycle, in particular INK4Bp15, and one of its downstream targets, Cdk6. Previously, it has been demonstrated that FUS was directly recruited to the regulatory region of the CCND1 gene, which encodes cyclinD1, by non-coding RNA (ncRNA) [58]. This recruitment leads to interference with the transcriptional complex formation and consequently to decreased cyclinD1 expression [27]. In our model the level of cyclinD1 transcript does not show any significant variation, differently from its interactor Cdk6, which instead shows a high reduction in its mRNA level. This suggests that FUS indirectly regulates the expression of cell cycle regulators, probably due to its control of miRNA processing [5,32,59].

One more scenario that could be explored with regard to the arrest of the cell cycle in G1, is the involvement of FUS in the control of DNA damage response [60,61]. At the molecular level the function of FUS in DNA damage repair is not yet clear. In response to DNA damage, FUS is rapidly recruited to DNA damage sites prior to H2AX phosphorylation, and interacts with HDAC1 [62]. FUS may be recruited to modulate chromatin conformation changes, stabilizing DNA damage repair complexes. The expression of WT-FUS protein, all localized in the nucleus, probably determines changes in its interaction with replicative complex, causing modifications in the DNA replication. When the concentration of cellular FUS returns to almost the physiological level the NSPCs can overcome the checkpoint G1/S and continue their proliferation [27,63].

On the other hand, the FUS mutation P525L drives to an abnormal subcellular distribution of the protein in the cytoplasm, where the main FUS functions in the transcriptional regulation are disrupted [64]. In fact, we observe a dramatic decrease of NSPC growth. Interestingly, the arrest of cell cycle progression is mediated by the signaling of INK4Bp15 and Cdk6, as detected for the human WT-FUS exogenous expression, suggesting the involvement of a mediator of the FUS activity on cell growth. The cyclin-dependent kinase inhibitor 2B (CDKN2B) gene encodes p15, which directly blocks the interaction of Cdk4/6 with cyclinD, similarly to p16 [65], lying close to tumor suppressor gene CDKN2A (that encodes INK4ap16), and is frequently dysregulated in human malignancies (reviewed in [66]). Dysregulation of CDKN2B has also been reported to be involved in the molecular mechanism of the apoptosis of smooth muscle cells [67,68]. By in silico analysis we found that CDKN2B is a virtual target of miR-15a-5p, one of the possible miRNAs regulated by FUS. This suggests that the regulation of miRNA processing is strongly involved in the neurogenesis of the spinal cord [69], probably controlling the expression of cell cycle regulators. Furthermore, our data indicate that the cell cycle arrest determined by the expression of mutant FUS is permanent, compared to the temporary effect mediated by the human WT-FUS. The transition from temporary to stable cell cycle arrest and the involvement of the CDKN2B may be considered the initial step of cellular senescence [70].

As it is known, many earlier studies showed that the constitutive overexpression of WT-FUS causes ALS-like phenotypes in vivo [18,19,71], and determines neuronal cell death via the mitochondrial apoptotic pathway [72]. All of these studies investigate the effect of very high levels of FUS in post-mitotic cells. Here, we report for the first time the effect of the expression of human WT-FUS or mutant FUS on NSPC properties, such as proliferation and differentiation. We studied the consequences due to the expression of both WT and mutant FUS on neuronal differentiation during the early committed stages. We showed that exogenous expression of FUS is anti-neurogenic. It is possible that WT-FUS expression preferentially drives NSPCs towards astrocytic lineage or, on the other hand, could kill the neurons that generate. Our data suggest that the expression of WT-FUS maintains NSPCs in an undifferentiated state, as indicated by the elevated expression of Nestin protein after ten days of differentiation. Furthermore, when we induced the expression of the transgene during proliferative but not under differentiative conditions, we were able to observe a higher induction of astrocytes instead of neuronal cells, suggesting that FUS interferes, directly or indirectly, with the expression of neuronal genes. Furthermore, the expression of mutant FUS itself drives the cells preferentially towards a glial lineage, strongly reducing the number of developing neurons. These data show that an unbalanced level of FUS during proliferation and at early differentiation stages may induce NSPCs in a quiescent state, slowing down all of their replicative and differentiative properties.

### Conclusions and Perspectives

In this study, we first analyzed the effect of elevated levels of human WT-FUS and one FUS ALS-linked mutation, associated with a juvenile and aggressive phenotype of the disease, in the regulation of neural stem cells’ proliferation and differentiation. We developed a cellular model based on murine NSPCs isolated from the spinal cord (one of the main regions affected by ALS), and engineered with inducible plasmids, allowing for a pharmacological and temporally regulated expression of hFUS. By using a gain-of-function approach, we demonstrated that either hWT-FUS or hP525L-FUS strongly impaired cell expansion and neuronal differentiation capabilities, indicating that FUS is an important regulator of NSPC properties. Further studies based on loss-of-function approaches (FUS knock out or knock down) will help to elucidate FUS function in NSPCs.

We believe that this study increases our knowledge on FUS function in cells of neural origin that do not show any sign of transformation and provides an excellent in vitro model to test how FUS is involved in neurodegeneration. NSPC lines expressing, in a temporally regulated manner, high levels of hWT-FUS (a condition that has been linked with ALS [18,19]), can represent a powerful tool to shed new light on the cell autonomous and non-cell autonomous mechanisms driving neuronal degeneration, e.g., in patients carrying FUS mutations in the untranslated region of this gene. However, in order to better understand the relation between FUS mutations and ALS disease progression it will be crucial to adopt human cellular models. Human NSPC lines, isolated from the fetal spinal cord, have already been produced in our laboratory [20] and can be used to assess FUS function both in NSPCs and in neuronal differentiated cells. The results could have important implications in the understanding of FUS biology and disease, with reference to ALS-linked disease.

## 4. Materials and Methods

### 4.1. Materials

All cell culture media, supplements and antibiotics were purchased from Sigma-Aldrich (St. Louis, MO, USA).

### 4.2. Cell Cultures

Neural stem progenitor cells (NSPCs) were isolated from 4–5 mouse embryonic spinal cords at E13.5 and adapted to cell cultures as previously described [33]. Cells were maintained in basal medium (DMEM/F12, 1% penicillin/streptomycin, 0.1M L-glutamine, 23.8 mg/100 mL Hepes, 7.5% NaHCO3, 0.6% glucose), supplemented with 20 ng/mL of human recombinant epidermal growth factor (EGF; R&D), 10 ng/mL of basic fibroblast growth factor (bFGF; Peprotech, Rocky Hill, NJ, USA), and 1% homemade N2 supplement. This medium is termed Expansion Medium. NSPCs were routinely expanded in T25 flasks (Corning Inc., Corning, NY, USA) that were coated with 10 µg/mL poly-ornithine and 5 µg/mL laminin, and were cultured at 37 °C in a 5% CO_2_ atmosphere. Cells were passaged every 3–5 days using Accutase and usually seeded at a density of 10,000–15,000 cells/cm^2^. For neuronal differentiation NSPCs were plated in Expansion Medium (18,000 cells/cm^2^) and 24 h later Expansion Medium was replaced with basal medium containing 10 ng/mL of bFGF, 1% N2, 2% B27, and 0.5 nM DAPT (Tocris Bioscience, Bristol, UK); this was termed Neuronal Differentiation Medium. Three days later the medium was partially replaced with fresh medium. After about 6 days under these conditions, NSPCs differentiated into a mixed culture of neurons (around 50%) and glial or residual progenitor cells [42]. For proteasome inhibition experiments, NSPCs were plated in Expansion Medium for 48 h (13,000 cell/cm^2^). After 2 h doxycline was added to the media to induce the transgene expression. The media was freshly replaced and the proteasome inhibitor MG132 (Z-Leu-Leu-Leu-al, Sigma-Aldrich) was added at a final concentration of 0.2 µM. The cells were collected after 24 h and analyzed by Western blotting.

### 4.3. Electroporation of NSPCs 

Cells at early passages were transfected using the Amaxa system according to manufacturer’s instructions (Nucleofector; Lonza, Basel, Switzerland). A total of 2 × 10^6^ cells were resuspended in 100 μL of an optimized NSC electroporation solution containing 2 μg of the plasmid epB-Puro-TT (provided by Prof. Irene Bozzoni [11]), containing the coding sequence of three different genes: epB-Puro-TT-RFP containing the coding sequence of Red Fluorescent Protein, represents the control plasmid; epB-Puro-TT-FUS-P525L containing the coding sequence of human FUS gene carrying the substitution P525L; epB-Puro-TT-FUS-WT containing the coding sequence of human WT FUS gene. The three genes of interest were cloned under a Tet-responsive promoter, in a bicistronic plasmid also containing the Tet element under a promoter activated by doxycycline. Each plasmid was electroporated independently, generating 3 independent cell lines. Transgene expression was controlled by a promoter inducible by adding doxycycline (50 ng/mL) to the culture medium. In order to increase integration efficiency, plasmids were co-transfected with a second plasmid helper containing the coding sequence of an integrase. After electroporation, 40,000 cells/cm^2^ were plated on poly-ornithine/laminin-coated flasks in pre-warmed and carbon dioxide equilibrate expansion medium. One day after transfection NSPCs with stable integration of the transgene were selected with puromycin (1 μg/mL) for two weeks. Under these experimental conditions we measured, by ICC, a detectable level of transgene expression in around 70% of the cells.

### 4.4. Immunocytochemistry

Cells were plated on glass coverslips coated with poly-ornithine/laminin. At the appropriate time points, cells were fixed with 4% of paraformaldehyde (PFA, Sigma-Aldrich) in PBS for 20 min. After fixation, cultures were washed with PBS pH 7.4, and then incubated in PBS containing 5% of the appropriate normal serum and 0.025% Triton X-100 (blocking solution) for 45 min at RT. Primary antibodies, listed below, were then applied and incubated overnight at 4°. Secondary Alexa Fluor or Cy3 antibodies, in the presence of 10 μg/mL of Hoechst 33342 (Thermo Fisher, Waltham, MA, USA), were applied for 2 h at RT. After rinsing with PBS, cells were coverslipped with DAKO mounting medium (Agilent Technologies, Santa Clara, CA, USA). The following primary antibodies were used: monoclonal anti-Nestin (1:1000; Millipore, Burlington, MA, USA), polyclonal anti-MAP2 (1:300; Millipore), polyclonal anti-GFAP (1:500; Agilent Technologies), monoclonal anti-Ki67 (1:500; Leica Biosystems, Wetzlar, Germany), monoclonal anti-FUS (1:500; Santa-Cruz Biotechnology, Dallas, TX, USA), polyclonal anti-FLAG (1:500; Sigma-Aldrich), monoclonal anti-FLAG M2 (1:500; Sigma-Aldrich). Immunofluorescence was observed using Nikon Eclipse TE300 microscope. A total of 9 random fields from each coverslip were photographed by Nikon digital camera DS-U3 and counted. Nuclear DNA was counter-stained with Hoechst 33342. At least 400 cells per experiment were counted and each experiment was repeated from three or more separate cultures. The results are expressed as mean ± SEM [13,25].

### 4.5. RNA Extraction

RNA was extracted by using a modified version of a previously described procedure [73]. Total RNA from cell cultures was prepared by TriReagent extraction procedure (#T9424; Sigma-Aldrich), following manufacturer’s instructions. Briefly, the cells were lysed in the appropriate volume of cold Tri-Reagent for 5 min and separated in three phases by adding chilled chloroform and by centrifuging at 12,000 g. The upper aqueous phase was transferred to a clear pre-chilled tube and the RNA was precipitated for 40 min, in cold isopropyl alcohol, and then centrifugated at 12,000 g. The obtained RNA pellets were washed 2 times with ice-cold 75% ethanol and upon centrifugation at 7500 g resuspended in 50 µL of RNase-free water. The RNA yield was determined with NanoDrop2000 Spectrophotometer (Thermo Fisher Scientific, Waltham, MA, USA).

### 4.6. Real-Time RT-PCR and Quantification of Gene Expression

For each RNA sample, 1 µg of total RNA was reverse-transcribed using QuantiTect Reverse Transcription (#205311; Qiagen, Hilden, Germany), allowing for an effective removal of contaminating genomic DNA and reverse transcription, following the manufacturer’s instructions.

cDNA amplification was performed on a RotorGene Q PCR cycler (Qiagen) using the QuantiFast SYBR Green PCR kit, following the manufacturer’s instructions. Each PCR reaction, for a final volume of 20 µL, contained 2 µL of cDNA (corresponding to 50 ng of total RNA) template, 0.5 µM forward primer, and 0.5 µM reverse primer. Each PCR assay consisted of 2 min at 95 °C for enzyme activation and 40 cycles of denaturation at 95 °C for 30 s, and annealing/extension at 60 °C for 10 s. The specificity of the PCR reactions was confirmed by melting curve analysis [74].

Data were calculated using the CT method [75] and expressed as relative quantities after β-actin normalization. Results were obtained from samples run in triplicate from three or more independent experiments. Primer sequences for the examined genes were designed with Primer3 program (http://bioinfo.ut.ee/primer3, accessed on 15 July 2021) and are listed below:
FUS humanF: GCT GCC ATC ACA AGC ATA GC R: CAG CCT GGA TGA CAG AGC AAFUS mouseF: CTG GAA CTT TGT TGC TTG GCG R: CCC CGT AGC TTT GAG TTG CTT Gbeta-actinF: CCC AGG CAT TGC TGA CAG G R: GCT GGA AGG TGG ACA GTG AGp27F: GGG TTC CAG CTT GTT GTG TT R: GGC CAT TTT CCA TCT CTG AACD1F: CAG CTT GCT AGG GAA CTT GC R: GAG TCT CCG GTG CAT CAT TTp15F: CCT TCC CCT GTG AAC TGA AA R: GTG AAT CCC CAC ACA TGA CACdk4F: GGC CCT CAA GAG TGT GAG A R: CAT CAG CCG TAC AAC ATT GCdk2F: AGA CTC CTG CTG CCA CTG TT R: CCC TCT TGC TTG GTT CTG AGp16F: CAT CTG GAG CAG CAT GGA GTC R: GGG TAC GAC CGA AAG AGT TCGp57F: TTG AGT AAA AGC CCC CAC A R: TGG GCA GTA CAG GAA CCA TCdk6F: ACT CTC ACC TGC CAT CCA A R: GAG GCT TGC TTA GGG GAC T

### 4.7. Cell Growth

NSPCs were seeded in 6-well plates at the density of 13,000 cells/cm^2^ in Expansion Medium. After 2 h, doxycycline was added to the medium to induce the expression of the transgene. At the desired time points the cells were detached with Accutase and recovered in PBS. Cell counting was performed as previously described [76]. Briefly, 90 µL of cell suspension was mixed with 10 µL of 0.4% Trypan blue solution (Invitrogen, Waltham, MA, USA) and the viable (non-positive) and non-viable (positive for Trypan blue [77]) cells were counted in disposable Glasstic KOVA chamber (#87144E; KOVA International, Garden Grove, CA, USA), following manufacturer’s instructions.

### 4.8. MTT Assay

Cells were plated in technical triplicate in a 24-well plate in Expansion Medium and after 2 h the transgene expression was induced by adding doxycycline. At the specified time points, the media from each well were removed and replaced with Locke’s Solution (154 mM NaCl, 5.6 mM KCl, 3.6 mM NaHCO_3_, 2.3 mM CaCl_2_·2H_2_O, 5 mM HEPES, 5.6 mM Glucose, 2 mM Glycine) supplement with 0.5 mg/mL MTT (3-[4,5-dimethylthiazol-2-yl]-2,5-diphenyl tetrazolium bromide). After 1 h of incubation at 37  °C, 500 µL of HCl 0.04 M isopropyl alcohol was added to each well to solubilized cells [78]. Slight agitation for 10 min was followed by OD quantification at 570 nm. Results are expressed as relative to untreated controls using the following equation: (ODtreated–ODblank)/(ODuntreated—ODblank). The ODblank is determined by wells containing MTT and tissue culture media without cells.

### 4.9. Cell Cycle Analysis

NSPCs were seeded on poly-ornithine (10 µg/mL) laminin (5 µg/mL)-coated T25-flasks at the density of 16,000 cells/cm^2^ and maintained in Expansion Medium for the specific time points of analysis, in the presence or not of doxycycline. Determination of NSPC distribution among G0/G1, S, G2/M cell cycle phases was performed according to flow cytometry standard procedures previously used by us and other groups [76,79]. Briefly, at the specified time cells were collected in 500 μL PBS and fixed by the dropwise addition of 500 μL of cold (−20 °C) methanol whilst gently mixing, before being stored at 4 °C overnight. Following overnight fixation, the methanol-PBS 1:1 solution was removed by pelleting the cells and washing with PBS. The cells were resuspended in PBS containing 50 µg/mL propidium iodide (Sigma-Aldrich), 100 µg/mL RNase A (Sigma-Aldrich) and 0.1% Triton X-100, 10 μL propidium iodide stock solution for each sample and incubated in the dark at 37 °C for 20 min. Cell cycle distribution analysis based on propidium iodide labelling of DNA content was performed by an Epics XL (Beckman Coulter, Cassina De’ Pecchi, Milano, Italia) flow cytometry apparatus equipped with a laser at 488 nm wavelength excitation. For each sample 10,000 events were acquired and cell cycle monoparemetric histograms generated using the WinMDI 2.9 software (Purdue University Cytometry Laboratories, West Lafayette, IN 47907, USA).

### 4.10. BrdU Incorporation

NSPCs were plated on poly-ornithine (10 µg/mL) laminin (5 µg/mL) coated glass coverslips (Menzel-Glaser, Thermo) at the density of 8000 cells/cm^2^ in expansion medium, and supplemented, 2 h later, with doxycycline to induce the transgene expression. In order to assess cell proliferation in cells expressing or not human FUS, we took advantage of BrdU cell labeling, according to procedures previously described [43,80,81]. Briefly, parallel cultures treated with or without doxycycline for 48, 72 and 96 h, respectively, were incubated with 10 μM BrdU (Sigma-Aldrich) for 30 min, and then cells were fixed with 4% PFA for 20 min and processed. Briefly, cells were incubated with 2 M HCl for 1 h to obtain DNA denaturation, rinsed three times with PBS and pre-incubated with a blocking solution containing 0.1% triton X-100 and 5% normal rabbit serum. Detection of the BrdU-incorporating cells was performed according to standard immunocytochemistry procedures by using a primary rat antibody anti-BrdU (1:100, ListarFish; Cernusco sul Naviglio, Italy) and the specific polyclonal rabbit Cy3 conjugated secondary antibody (Jackson Immunoresearch, West Grove, PA, USA). The percentage of cells positive for BrdU was determined by counting the number of BrdU-positive cells in 9 different and randomly chosen microscopic fields in each of three coverslips per condition. Total cell number for each analyzed microscopic field was determined by counting Hoechst 33342-stained cells. Results are the mean of at least three independent experiments.

### 4.11. Western Blotting

To obtain whole cell extract, NSPCs were harvested in RIPA buffer containing 320 mM sucrose, 50 mM TRIS pH 7.5, 1% Triton, 10% glycerol and 1% of inhibitor of proteases cocktail (Sigma-Aldrich), incubated on ice for 30 min and centrifuged for 12 min at 13,000× *g*. Protein amount was quantified by Bradford assay (BioRad, Hercules, CA, USA) and 10 or 20 µg of protein was subjected to SDS-polyacrylamide gel electrophoresis and transferred overnight onto polyvinylidene fluoride membrane (Immobilion-PSQ; Millipore) [82]. Membranes were blocked in 5% non-fat dry milk and then incubated with the following primary antibodies: monoclonal anti-FUS (1:750; Santa-Cruz), polyclonal anti-FLAG (1:750; Sigma-Aldrich), monoclonal anti-PCNA (1:750; Sigma-Aldrich), polyclonal anti-Cdk2 (1:400, Santa Cruz), polyclonal anti-DCX (1:200, Abcam, Cambridge, UK), and polyclonal anti-PAX6 (1:200; BioLegend). Blots were washed and incubated with the appropriate horseradish peroxidase secondary antibodies (1:10.000, Jackson Immunoresearch). Blots were then washed again, incubated in lumi-light enhanced chemiluminescence substrate (BioRad) and exposed to chemidoc (BioRad). Densitometric analysis on scanned blots was performed using the ImageLab program (BioRad).

### 4.12. Statistical Analysis

Comparisons were performed using unpaired two-tailed Student’s *t*-test and Mann–Whitney test. Data are the average of three or more independent experiments and are presented as the mean ± SEM. Differences are considered significant at *p* < 0.05.

## Supplementary Materials

The following are available online at https://www.mdpi.com/article/10.3390/ijms22147566/s1.

## Abbreviations

Amyotrophic lateral sclerosis (ALS), Fused in Sarcoma/Translocated in Liposarcoma (FUS/TLS), neural stem progenitor cells (NSPCs), frontotemporal lobar degeneration (FTLD), human wild-type FUS (hWT-FUS), Cyclin D1 (CCND1), cyclin dependent kinase inhibitor 1B (CDKN1B/p15), benzyloxycarbonyl-leucyl-leucyl-leucinal (MG132), ubiquitin proteasome system (UPS), 3-(4,5-dimethylthiazol-2-yl)-2,5-diphanyltetrazolium bromide (MTT), doxycyclin (DOXY), N-N-(3,5-Difluorophenacetyl)-L-alanyl-S-phenylglycine t-butyl ester (DAPT), doublecortin (DCX), microtubule-associated protein 2 (MAP2), cyclin-dependent kinase (CDK), glial fibrillary acidic protein (GFAP), bromodeoxyuridine (BrdU), inhibitors of CDK4 (INK4), histone deacetylases (HDAC), Cyclin Dependent Kinase Inhibitor 2B (CDKN2B), Dulbecco’s Modified Eagle’s Medium (DMEM), epidermal growth factor (EGF), basic fibroblast growth factor (bFGF), proliferating cell nuclear antigen (PCNA).
