# Supplementary material for: Wild-Type and Mutant FUS Expression Reduce Proliferation and Neuronal Differentiation Properties of Neural Stem Progenitor Cells
Source: Int J Mol Sci. 2021 Jul 15;22(14):7566. doi: 10.3390/ijms22147566 (PMC8304973; doi:10.3390/ijms22147566)
Supplement: Supplementary file 1 [file ijms-22-07566-s001.zip › ijms-1232651-supplementary.pdf]

Supplementary material

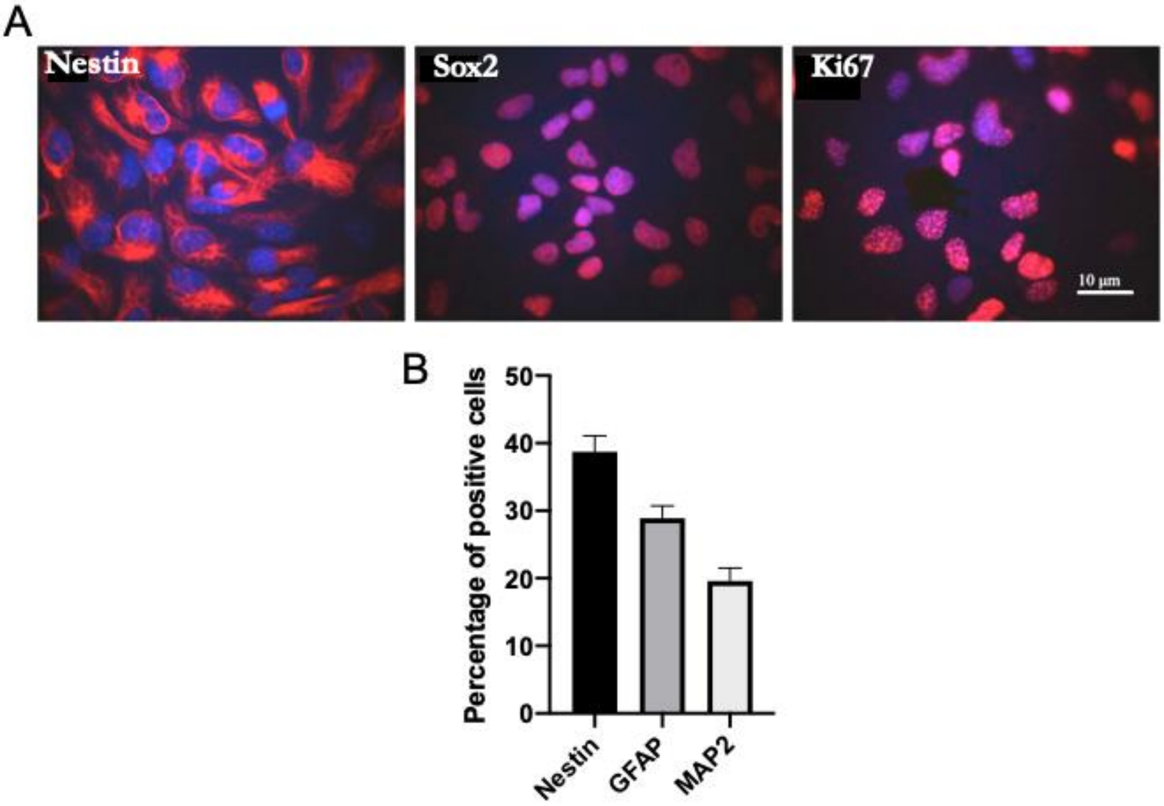

Supplementary Figure S1. NPSCs isolated from spinal cord retain their stemness properties.

A) Representative images of NSPCs immunopositive for two well-known stem cell markers (Nestin and Sox2) and for the proliferative marker Ki67. Note that virtually all the NSPCs maintained in expansion medium are immunopositive for the neural markers Nestin and Sox2.

B) Quantification of NSPC differentiation capability upon growth factor removal and differentiation for 3 days. The cells are able to differentiate in a mixed population containing astrocyte-like cells (GFAP positive) and neurons (Map2 positive), indicating that they are multipotent in our culture conditions.

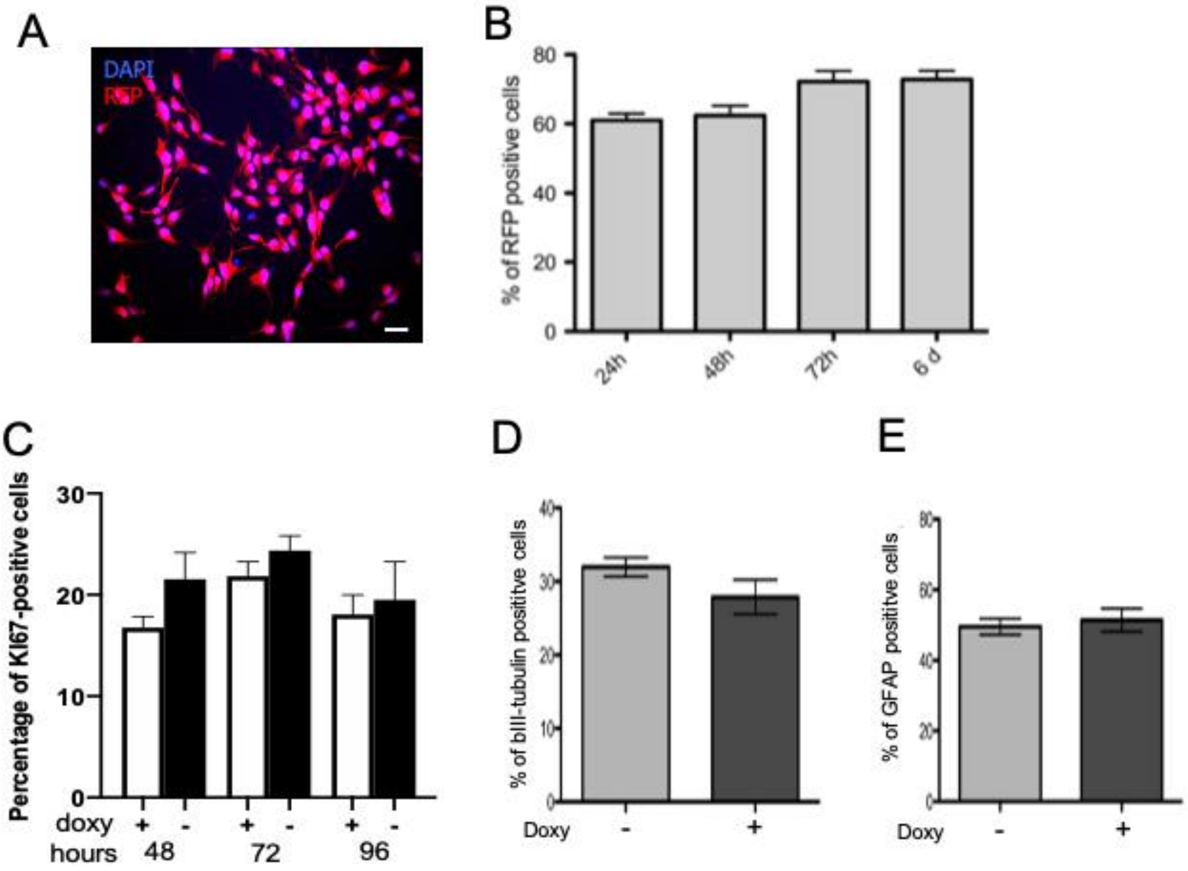

Supplementary Figure S2: RFP-NSPC properties are not affected by doxycycline treatment.

A-B) Representative image of RFP-NSPCs treated with doxycycline (**A**; Scale bar 30μm) and quantification of the percentage of RFP-expressing cells at the different time points analyzed (**B**). RFP is already expressed after 24 hours of doxycycline treatment and its expression remains substantially unchanged with time. **C)** The proliferative marker Ki67 is quantified in RFP-NSPCs maintained in Expansion Medium between 48 to 96 hours showing that the doxycycline administration does not affect the proliferation rate of NPSCs. **D-E)** RFP-NPSC capacity to generate neurons (D) and glia (E) is not affected by doxycycline treatment.
